# Prognostic value of IMP3 immunohistochemical expression in triple negative breast cancer

**DOI:** 10.1097/MD.0000000000019091

**Published:** 2020-02-14

**Authors:** Nikoleta Sjekloča, Snjezana Tomić, Ivana Mrklić, Filip Vukmirović, Ljiljana Vučković, Ingrid Belas Lovasić, Marina Maras-Šimunić

**Affiliations:** a Medical Faculty, University of Montenegro, Montenegro; b Department of Pathology, Forensic Medicine and Cytology, University Hospital Split; c School of Medicine, University of Split; d Department of Pathology, Clinical Center of Montenegro, Montenegro; e Department of Oncology, Clinical Hospital Center Rijeka; f Department of Diagnostic and Interventional Radiology, University Hospital Split, Croatia.

**Keywords:** basal morphology, IMP3, TNBC, tumor size, vascular invasion

## Abstract

Triple negative breast cancer (TNBC) account for 12% to 17% of all breast cancers. It is a heterogeneous group of tumors associated with aggressive clinical course. Insulin-like growth factor II mRNA binding protein 3 (IMP3) belongs to a family of insulin-like growth factor type II (IGF2), which plays a key role in the transmission and stabilization of mRNA, cell growth, and migration during embryogenesis. Increased expression of IMP3 is associated with aggressive behavior of different tumor types, advanced clinical stage, distant metastasis, and shorter overall survival (OS).

The study included 118 patients with breast carcinoma diagnosed as TNBC and immunohistochemical staining for estrogen receptors (ER), progesterone receptors (PR), epidermal growth factor receptor 2 (HER2/neu), Ki-67, and IMP3 was performed. Correlations between categorical variables were studied using the chi-square and the Mann–Whitney U test. For survival analysis, the Kaplan–Meier method, log-rank test and the Cox proportional hazard regression model were used.

Positive expression of IMP3 protein was present in 35.6% of TNBC. The presence of basal morphology was observed in 46.6% of TNBC. Positive IMP3 expression was connected with larger size of tumor, higher clinical stage, and basal morphology (*P* = .039, *P* = .034, *P* < .001). Disease-free survival and OS were significantly shorter in IMP3 positive TNBC.

According to results of our study IMP3 expression can be used as negative prognostic factor for triple negative breast carcinomas. Targeting IMP3 molecule could be an effective approach to the management of a triple negative breast cancer with new immunological therapies, which does not yet exist for this group of tumors.

## 1 Introduction

Triple negative breast cancer, accounting for 12% to 17% of all breast cancers, is a heterogeneous group of tumors associated with aggressive clinical course, blood borne liver, lung, and brain metastasis while metastases in loco regional lymph nodes are less common in comparison to other breast tumors types.^[1–4]^ Despite good initial response to neoadjuvant chemotherapy protocols, patients with this type of tumor have higher rates of distant metastases and ultimately poor prognosis.^[1,2,5]^ Recently, substantial efforts were made toward improving treatment outcome for TNBC patients, requiring further subclassification based on prognostic value of new molecular biomarkers.

Insulin-like growth factor II mRNA binding protein 3 (IMP3) belongs to a family of insulin-like growth factor type II (IGF2), which plays a key role in the transfer and stabilization of mRNA, cell growth, and migration during embryogenesis. To date, there are three known members of IMP3 family proteins: IMP1, IMP2, and IMP3^[6]^ Expression of IMP3 is negative in normal, mature tissues, but was found as positive in the malignant tumors of the colon, kidney, bladder, pancreatic ductal adenocarcinoma, gastric cancer, non-small cell lung cancer, melanoma, thyroid cancer, osteosarcoma, and breast cancer and related with aggressive behavior of the tumor, advanced clinical stage, and distant metastases.^[7–18]^

Recent studies have shown that IMP3 expression is closely associated with estrogen receptor negativity and positive expression of EGFR.^[19]^ IMP3 acts as a promoter of aggressive behavior in triple negative breast cancer and contributes to breast cancer chemo resistance.

IMP3 binds to breast cancer resistance protein (BCRP) mRNA and regulates BCRP expression. BCRP, also known as ATP-binding cassette super-family G member 2 , is a member of the ATP-binding cassette (ABC) transporters and a major effector of resistance to doxorubicin and mitoxantrone in breast cancer. Depletion of IMP3 expression in triple-negative breast cancer cells increased significantly their sensitivity to doxorubicin and mitoxantrone.^[20,21]^

## 2 Materials and methods

### 2.1 Patients

In our study, 118 patients with triple negative breast cancer, undergoing surgery between January 2003 and December 2009, who did not receive preoperative chemotherapy and had available paraffin embedded tissue blocks were reviewed retrospectively. Clinical information was collected through the breast cancer database of the Department of Oncology and Radiotherapy, Split University Clinical Center.

All histological and Immunohistochemistry (IHC) tumor slides were evaluated by two pathologists (ST, IM) and graded according to Elston and Ellis grading method.^[22]^ Histological types were determined according to WHO and staged according to TNM Classification.^[23,24]^

Ethical committee for Biomedical Research of the Clinical Hospital Center Split approved that this research are in compliance with the Helsinki Declaration (reference number 49-1/06).

### 2.2 Immunohistochemical analysis

Sections from fixed, paraffin embedded, cancer tissues were stained by hematoxylin/eosin with additional immunostains for ER (1:200, Dako, Glostrup, Denmark), PR (1:100, Dako), epidermal growth factor receptor 2 (HER2/neu) (HercepTest assay, Dako), Ki-67 (1:200, Dako), and IMP3 (1:150, Dako).

**Figure 1:**
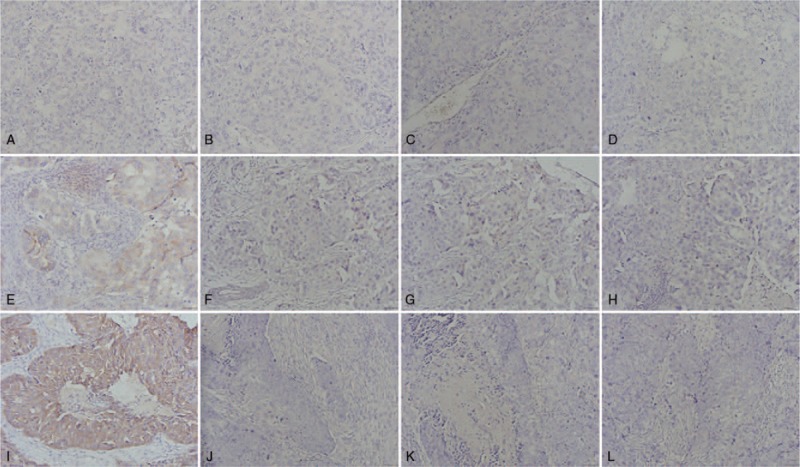
Immunohistochemical analysis: (A) negative IMP3 staining, (B) negative ER staining, (C) negative PR staining, (D) negative HER2 staining, (E) weak positive IMP3 staining, (F) negative ER staining, (G) negative PR staining, (H) negative HER2 staining, (I) strong positive IMP3 staining, (J) negative ER staining, (K) negative PR staining, and (L) negative HER2 staining. ER = estrogen receptors, HER2 = epidermal growth factor receptor 2, IMP3 = insulin-like growth factor II mRNA binding protein 3, PR = progesterone receptors.

Immunoassays were performed on Ventana BenchMark Ultra autostainer (Roche, Tucson, AZ). HER2 status was evaluated by IHC (Hercept Test, Dako, Glostrup, Denmark) or by chromogenic in situ hybridization (SPOT-Light HER2 CISH Kit, Invitrogen/Zymed, Camarillo, CA). Tests were scored according to ASCO/CAP guidelines.^[25]^ ER and PR were considered positive if at least 1% of the invasive tumor cell nuclei were positive (Fig. 1).^[4]^

IMP3 staining was evaluated semiquantitatively according to finding of Brown staining in the cytoplasm. The intensity of staining was estimated as: absent (0), weak (1), moderate (2), and strong (3) cytoplasmic staining. The percentage of stained cells was scored: 0% (0), 1% to 25% (1), 25% to 50% (2), 51% to 75% (3), and 75% to 100% (4). Based on the sum of the score obtained by evaluation of the intensity of staining and the percentage of stained cells the final sum was formed, and interpreted in the following way: 0 to 1 as negative staining, 2 to 4 as weak positive staining, and 5 to 7 as strong positive staining (Fig. 1).

**Figure 2:**
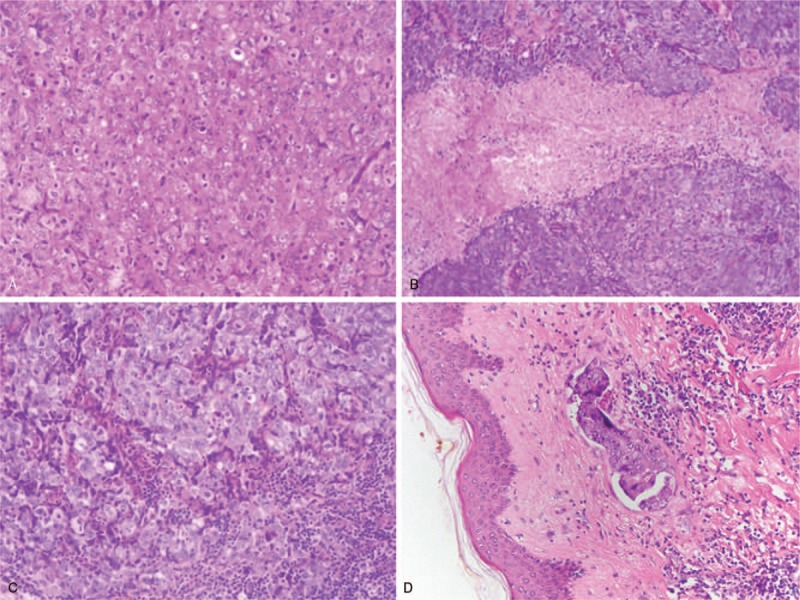
(A) syncytial growth pattern, (B) large central acellular/necrotic zone, (C) dense lymphocytic infiltrate at the periphery of the invasive component, and (D) vascular invasion.

Basal like (BL) morphology was considered positive if characteristic features, such as syncytial growth pattern, high-mitotic index, large central acellular/necrotic zone, pushing borders, dense lymphocytic infiltrate at the periphery of the invasive component, and the presence of metaplastic and medullary elements were present (Fig. 2).^[26]^

Vascular invasion was considered positive if the presence of tumor cells in endothelium confined spaces on the periphery of the tumor was found (Fig. 2).

Ki-67 was scored by counting 1000 tumor cells using the Olympus Image Analyser (magnification 400×), at the hot spots and at the periphery of the invasive component. Data are expressed as percentages of positive cells.^[27]^

### 2.3 Statistical analysis

Data were analyzed using Statistics for Windows Release 12.0 (Statsoft, Tulsa, OK). All *P*-values <.05 were considered statistically significant. All statistical tests were two-sided, with 95% confidence interval. Correlations between categorical variables were studied using chi-square test. For univariate analysis, survival time was analyzed by the Kaplan–Meier method and the log-rank test was used to assess differences among groups. For disease-free survival (DFS) and overall survival (OS), survival time was censored at death, if the cause was not breast cancer or if the patient was alive without relapse on March 1, 2011. For multivariate analysis Cox proportional hazard regression model was used to simultaneously examine all factors predictive of survival in univariate analysis.

## 3 Results

Out of 118 TNBC, 99 (83.9%) were invasive carcinomas not otherwise specified (NOS), while 19 (16.1%) were the other specific types. The majority of TNBC were histological grade 3 (80.5%). The presence of basal morphology was observed in 55 (46.6%) tumors. The presence of vascular invasion was found in 40 (33.9%) patients (Table 1).

**Table 1:**
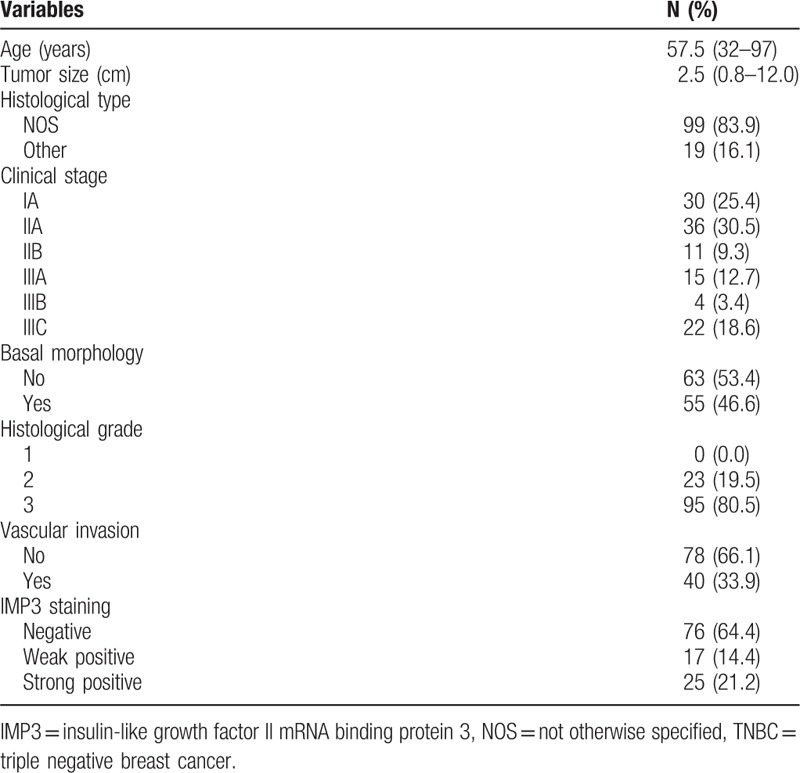
Clinicopathological features of 118 TNBC patients.

Out of 118 TNBC 42 (35.6%) tumors showed positive expression of IMP3. Statistical significance was found regarding the IMP3 expression and tumor size, clinical stage, and basal morphology (*P* = .039, *P* = .034, *P* < .001, respectively (Table 2).

**Table 2:**
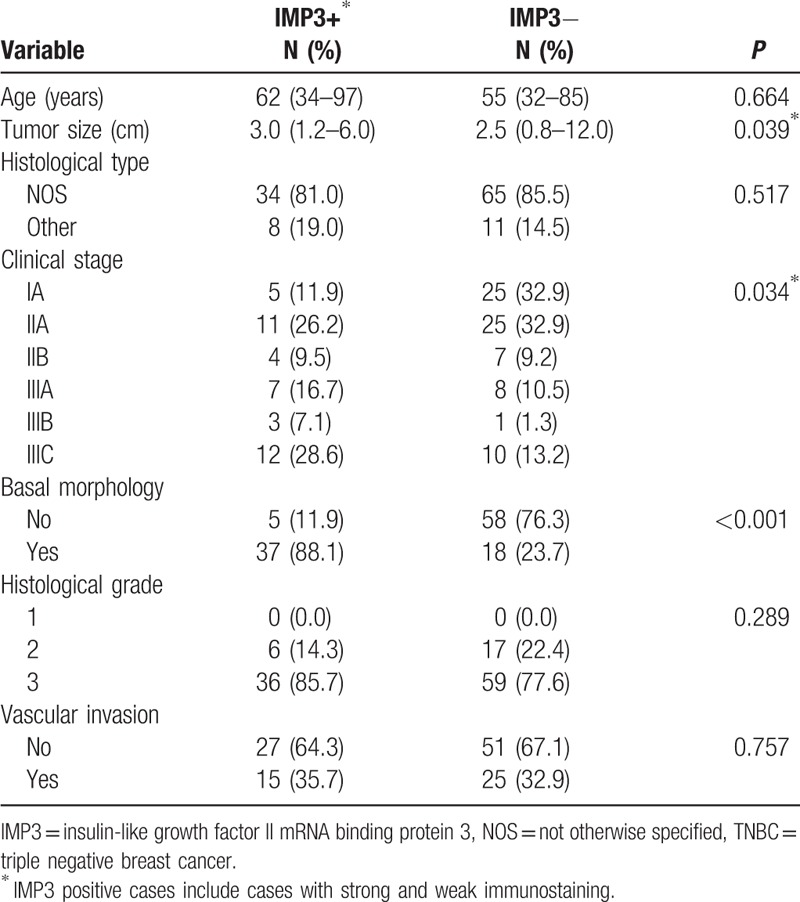
Correlation between IMP3 immunoexpression and clinicopathological parameters of 118 TNBC patients.

**Figure 3:**
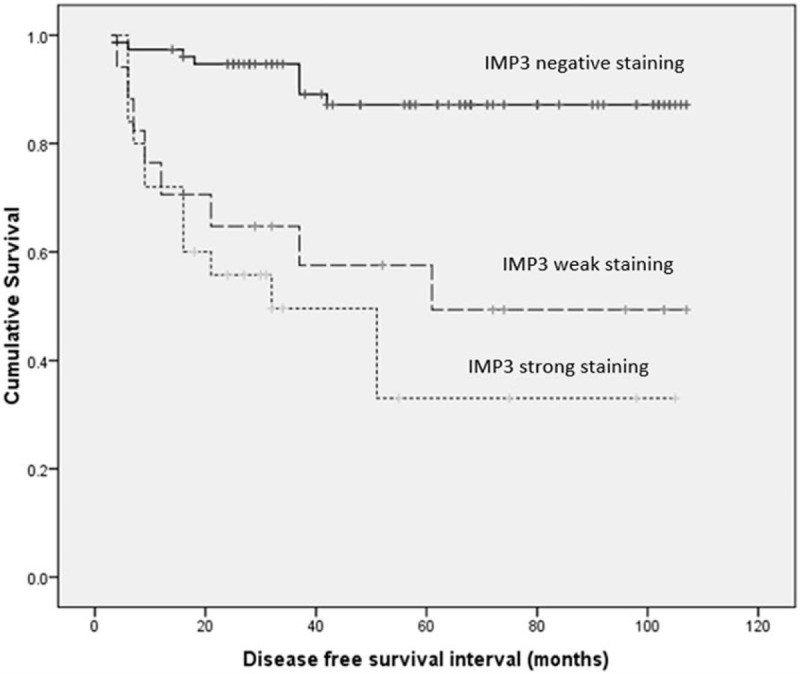
Kaplan–Meier curve for DFS interval for IMP3 (solid line: negative staining, dashed line: weak positive staining; dotted line: strong staining). DFS = disease-free survival, IMP3 = insulin-like growth factor II mRNA binding protein 3.

**Figure 4:**
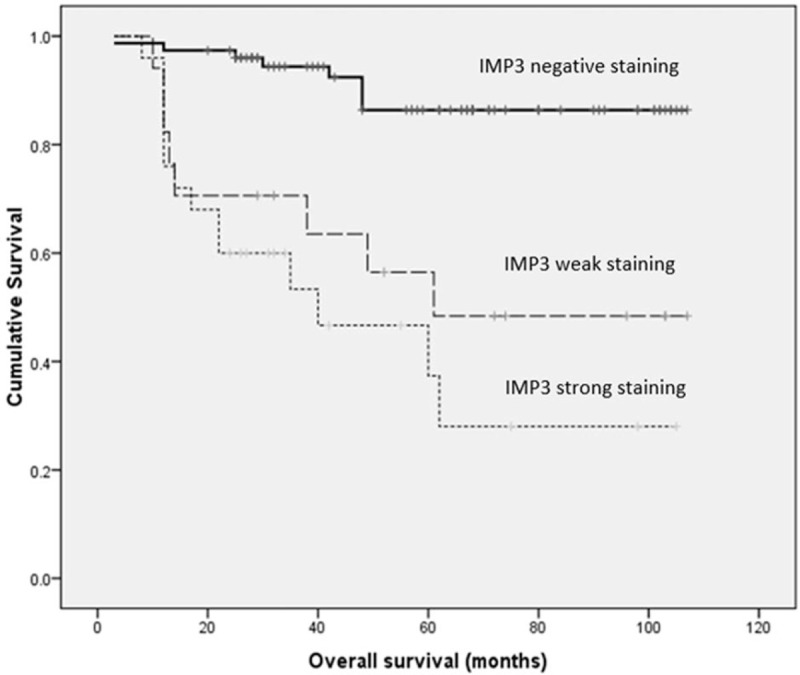
Kaplan–Meier curves of OS by IMP3 (solid line: negative staining; dashed line: weak positive staining; dotted line: strong staining). IMP3 = insulin-like growth factor II mRNA binding protein 3, OS = overall survival.

Univariate survival analysis revealed that age (*P* = .030), clinical stage (*P* < .001), basal morphology (*P* = .001), and IMP staining (*P* < .001) statistically correlated with DFS (Table 3) (Fig. 3). Univariate survival analysis revealed that age (*P* = 0.034), clinical stage (*P* < .001), basal morphology (*P* = .001), and IMP3 staining (*P* < .001) statistically correlated with OS (Table 4) (Fig. 4).

**Table 3:**
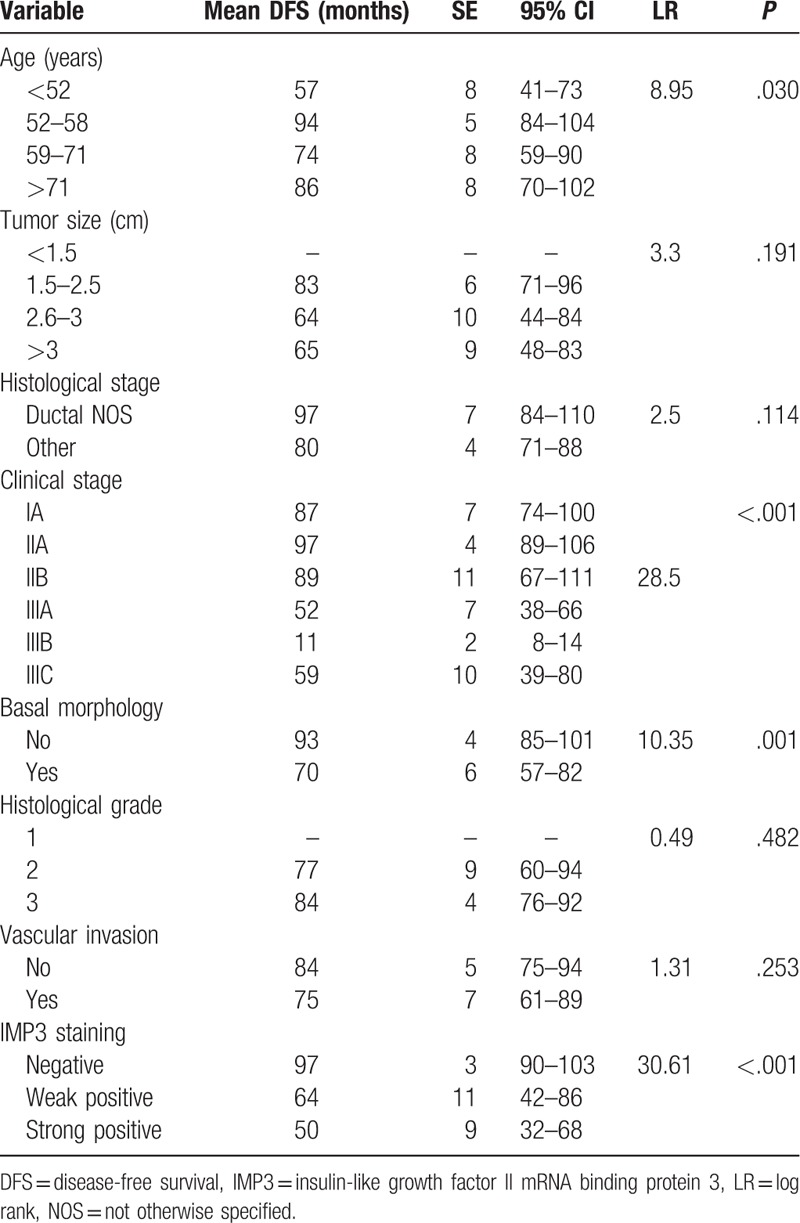
Analysis of survival Log rank test for DFS interval for the studied indicators.

**Table 4:**
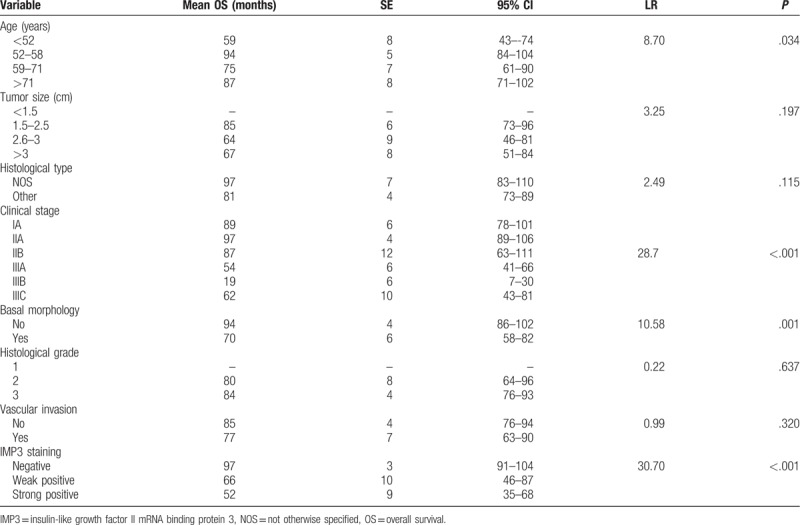
Analysis of survival Log rank test for OS for the studied indicators.

Multinominal analysis was performed which included all variables that yielded significant *P*-value by univariate analysis. Therefore, an independent prognostic relevance was found for tumor size, clinical stage, and IMP3 immunostaining.

Regarding DFS, statistically significant predictors were tumor size (RR = 1.64, *P* = .016), clinical stage (RR = 1.25, *P* = .049), and IMP3 staining (RR = 2.84, *P* = .001) (Table 5). Multinomial analysis revealed that significant predictors for OS were tumor size (RR = 1.60, *P* = .022) and IMP3 (RR = 2.67, *P* = .001) (Table 6).

**Table 5:**
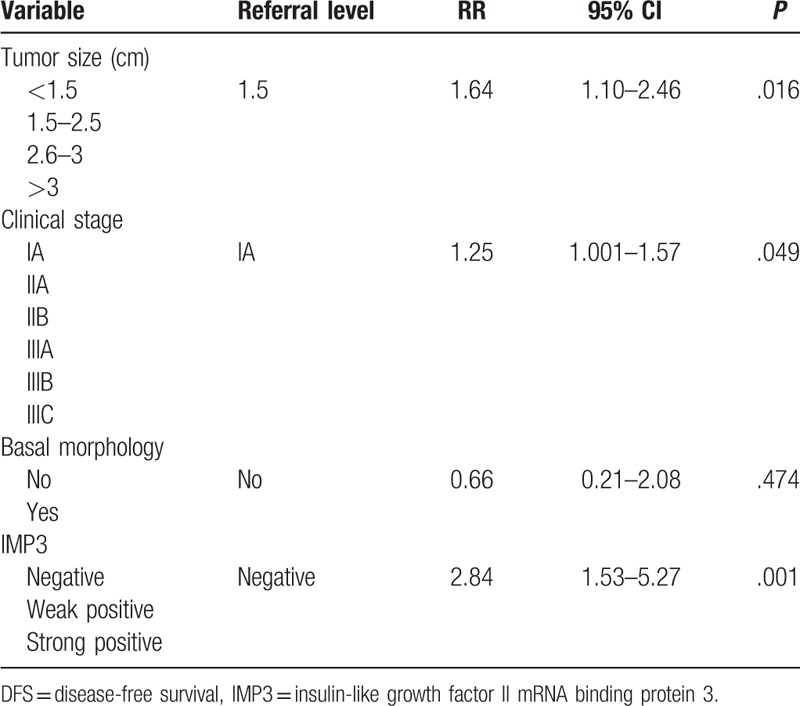
Multinominal Cox regression analysis for DFS interval.

**Table 6:**
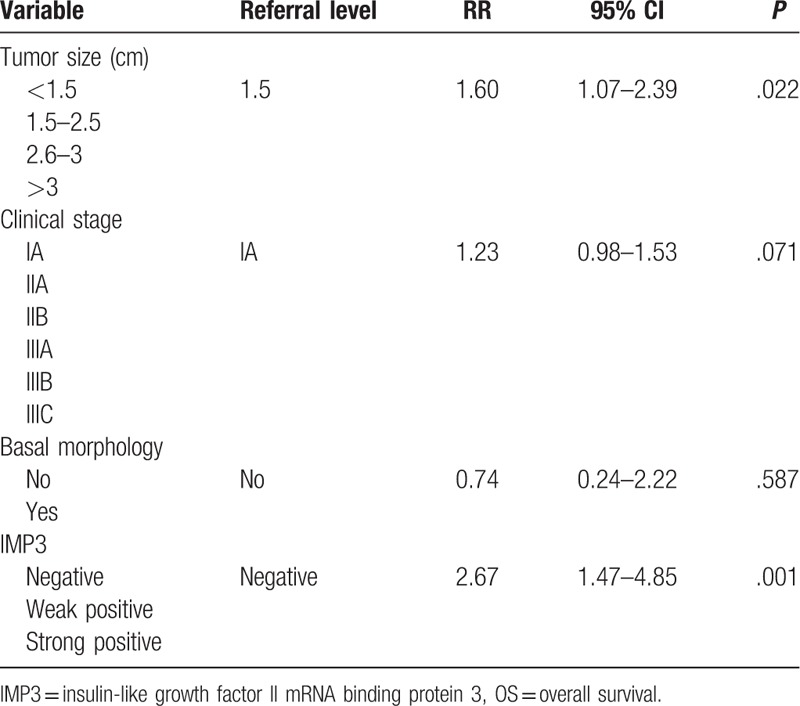
Multinominal Cox regression analysis for OS.

## 4 Discussion

TNBC represents a heterogeneous group of tumors characterized by aggressive tumor biology responsible for poor survival outcomes in comparison with other breast cancer types and lack of targeted therapies, which is creating high unmet need for better understanding of TNBC molecular nature and development of new subclassification of this disease.^[28]^ Discovering new biomarkers with potential prognostic and predictive value may trigger development of targeted therapies and ultimately improve TNBC outcome.

In our study the most common histological type was NOS (83.9%) and basal morphology was observed in 46.6% of TNBC, which is in concordance with results of previous studies.^[28–30]^ Out of 118 patients 42 (35.6%) had positive expression of IMP3, and this finding is in concordance with the research of Walter et al.^[31]^

Analysis of the relationship between IMP3 expression and histopathological parameters in our study revealed that positive IMP3 expression significantly correlated with greater tumor size, higher clinical stage and basal morphology which are clinical and pathological predictors of aggressive biologic behavior in breast cancer. The presence of vascular invasion was observed in 33.9% samples and there was no statistically significant correlation between vascular invasion and increased IMP3 expression. Mohammed et al have found that vascular invasion was more frequently present in TNBC versus non-TNBC tumors, as well in BL more than in non-BL ones.^[32]^ In our study, survival analyses have shown no correlation between vascular invasion and OS and DFS. Possible explanation for this finding is that in this particularly aggressive type of breast cancer vascular invasion is losing its prognostic value. The vast majority (85%) of the TNBC in our study was associated with high-histologic grade, only 19.5% tumors were moderately differentiated, and none of the samples belonged to well-differentiated category, which could be the explanation for lack of correlation between IMP3 immunoexpression and histologic grade.

Survival analysis that we performed revealed that DFS interval and OS in patients with triple negative breast cancer is shorter in cases of high-clinical stage, presence of basal morphology, and strong IMP3 immunostaining. Based on the Cox regression analysis, our study showed that increased expression of IMP3 is a negative predictive factor for the prognosis of triple negative breast cancer. According to our results, determination of IMP3 biomarker, may serve as independent prognostic factor within the heterogeneous group of triple negative breast cancer. In addition, IMP3 targeting could be efficient approach for TNBC management due to several reasons: IMP3 was not expressed in the normal tissue of the breast, the mechanism of action of this molecule is known (for the binding of sequence-specific RNA), and the inhibition of the expression of IMP3 molecule should increase the sensitivity of tumors to chemotherapy. IMP3 epitopes, based on their immunogenicity (i.e., the capacity to induce a strong and specific anti-tumor immune response), could be the target for the newly synthesized vaccine for the treatment of triple negative breast cancer.^[33–35]^ Cancer vaccines are designed to target only cancer cells and provide sparing of surrounding healthy tissue. The results of recent clinical trials have shown their safety and almost non-existing risk of autoimmune reactivity when being tested for the treatment of lung cancers and esophageal cancers; activating IMP3 specific T-cell immune response in patients with the HLA-A 24-positive carcinoma of the esophagus and lung.^[33,36,38]^

It is believed that the same mechanism of IMP3 specific T cell immune response represents a new possibility for the treatment of triple negative breast cancer.

## Author contributions

The work presented here was carried out in collaboration between all authors. Violeta Sjekloča and Snježana Tomić defined the research theme. Snježana Tomić and Ivana Mrklić performed pathohistological and immunohistochemical analysis and report. Filip Vukmirović, Ljiljana Vučković, Ingrid Belas Lovasić and Marina Maras-Šimunić participated in data collection and interpretation of statistical analysis. Violeta Sjekloča and Snježana Tomić performed literature review and wrote the paper. All authors were involved in drafting the manuscript or revising it critically for important intellectual content.
